# Effectiveness of neoadjuvant trastuzumab and chemotherapy in HER2-overexpressing breast cancer

**DOI:** 10.1007/s00432-013-1436-y

**Published:** 2013-04-20

**Authors:** Clara Natoli, Patrizia Vici, Isabella Sperduti, Antonino Grassadonia, Giancarlo Bisagni, Nicola Tinari, Andrea Michelotti, Germano Zampa, Stefania Gori, Luca Moscetti, Michele De Tursi, Michele Panebianco, Maria Mauri, Ilaria Ferrarini, Laura Pizzuti, Corrado Ficorella, Riccardo Samaritani, Lucia Mentuccia, Stefano Iacobelli, Teresa Gamucci

**Affiliations:** 1 Medical Oncology Unit, Department of Experimental and Clinical Sciences, University ‘G. d’Annunzio’, 66013 Chieti, Italy; 2 Division of Medical Oncology B, Regina Elena National Cancer Institute, 00144 Rome, Italy; 3 Unit of Biostatistics, Regina Elena National Cancer Institute, 00144 Rome, Italy; 4 Oncology Unit, Department of Oncology, Azienda Ospedaliera ASMN, Istituto di Ricovero e Cura a Carattere Scientifico, 42123 Reggio Emilia, Italy; 5 Oncology Unit I, Azienda Ospedaliera Universitaria Pisana, 56124 Pisa, Italy; 6 Oncology Unit, Nuovo Regina Margherita Hospital, 00153 Rome, Italy; 7 Medical Oncology, Sacro Cuore-Don Calabria Hospital, 37024 Negrar (VR), Italy; 8 Division of Medical Oncology, Department of Oncology, Belcolle Hospital, AUSL Viterbo, 01100 Viterbo, Italy; 9 Oncology Unit, Department of Oncology, S. Giovanni-Addolorata Hospital, 00184 Rome, Italy; 10 Oncology Unit II, Azienda Ospedaliera Universitaria Pisana, 56124 Pisa, Italy; 11 Medical Oncology, S. Salvatore Hospital, University of L’Aquila, 67100 L’Aquila, Italy; 12 Department of Oncology, “S.S Trinità” Hospital, 00039 Sora (FR), Italy

**Keywords:** Breast cancer, Pathological complete response, HER2, Neoadjuvant, Trastuzumab, Survival

## Abstract

**Purpose:**

Trastuzumab and chemotherapy is the current standard of care in HER2+ early or locally advanced breast cancer, but there are scanty literature data of its real world effectiveness.

**Methods:**

We retrospectively reviewed 205 patients with HER2+ breast cancer diagnosed in 10 Italian Medical Oncology Units between July 2003 and October 2011. All patients received neoadjuvant systemic therapy (NST) with trastuzumab in association with chemotherapy. Many different chemotherapy regimens were used, even if 90 % of patients received schemes including anthracyclines and 99 % received taxanes. NST was administered for more than 21 weeks (median: 24) in 130/205 (63.4 %) patients, while trastuzumab was given for more than 12 weeks (median: 12 weeks) in 101/205 (49.3 %) patients. pCR/0 was defined as ypT0+ypN0, and pCR/is as ypT0/is+ypN0.

**Results:**

pCR/0 was obtained in 24.8 % and pCR/is in 46.8 % of the patients. At multivariate logistic regression, nonluminal/HER2+ tumors (*P* < 0.0001) and more than 12 weeks of neoadjuvant trastuzumab treatment (*P* = 0.03) were independent predictors of pCR/0. Median disease-free survival (DFS) and cancer-specific survival (CSS) have not been reached at the time of analysis. At multivariate analysis, nonluminal/HER2+ subclass (DFS: *P* = 0.01 and CSS: *P* = 0.01) and pathological stage II–III at surgery (DFS: *P* < 0.0001 and CSS: *P* = 0.001) were the only variables significantly associated with a worse long-term outcome.

**Conclusions:**

Our data set the relevance of molecular subclasses and residual tumor burden after neoadjuvant as the most relevant prognostic factors for survival in this cohort of patients.

## Introduction

Preoperative or neoadjuvant systemic therapy (NST) for early or locally advanced breast cancer (LABC) is widely used to downstage the primary tumor, thus allowing a higher rate of conservative surgery (van der Hage et al. 2001; Semiglazov et al. 2011; Schott and Hayes 2012), with the same survival benefits as postoperative adjuvant chemotherapy (Bear et al. 2006; van der Hage et al. 2001; Fisher et al. 1997, 1998; Mauri et al. 2005). The human epidermal growth factor receptor 2 (HER2) oncogene is one of the most relevant prognostic and predictive factors for breast cancer patients. HER2 overexpression, which occurs in ~20 % of all breast cancers, has been associated with a poor prognosis both in the early and metastatic setting (Hudis 2007; Ross et al. 2009). However, HER2-positive (HER2+) tumors are highly chemo-sensitive (e.g., to anthracyclines and taxanes) and responsive to the humanized anti-HER2 monoclonal antibody trastuzumab (Herceptin^®^) (Brufsky 2010; Mariani et al. 2009). Adjuvant chemotherapy plus 52 weeks of trastuzumab is the current standard regimen for HER2+ early breast cancer (Costa et al. 2010; Garnock-Jones et al. 2010; Gianni et al. 2011). Trastuzumab in association with chemotherapy as NST, for early or locally advanced HER2+ breast cancer, has been extensively investigated in phase II–III clinical trials in the last few years (Anton et al. 2011; Burstein et al. 2003; Coudert et al. 2007; Dawood et al. 2007; Gianni et al. 2010; Penault-Llorca et al. 2007; Petrelli et al. 2011; Pierga et al. 2010; Robidoux et al. 2010; Ruiz et al. 2008; Sanchez-Munoz et al. 2010; Sikov et al. 2009; Untch et al. 2010, 2012; Wildiers et al. 2011; Buzdar et al. 2005, 2007; Horiguchi et al. 2009; von Minckwitz et al. 2010; Valachis et al. 2011). A recent systematic review shows that the addition of trastuzumab to chemotherapy has allowed to obtain rates of pathological complete response (pCR) significantly higher than chemotherapy alone (38 vs 21 %; *P* value <0.001) (Valachis et al. 2011), but a wide variability is observed in clinical trials, ranging from 12 to 67 % (Burstein et al. 2003; Valachis et al. 2011; Buzdar et al. 2005; Harris et al. 2007; Hurley et al. 2006).

Achievement of pCR after NST has been shown to be a good surrogate marker for superior long-term outcome, in terms of disease-free survival (DFS) and possibly overall survival (OS) (Fisher et al. 1998; Symmans et al. 2007; Mazouni et al. 2007; Kaufmann et al. 2006). However, this predictive potential of pCR has been recently questioned by various authors, particularly in relation to the molecular subclasses of breast cancer (Goldhirsch et al. 2011). In fact, patients with luminal A [steroid hormone receptors positive, HER2 negative, low proliferative activity] breast cancer usually show a very low rate of pCR, but their prognosis remains good even when no pCR is achieved (Angelucci et al. 2012; Colleoni et al. 2009; Huober et al. 2010; Precht et al. 2010; Straver et al. 2010; Kim et al. 2010; von Minckwitz et al. 2012). It has been recently reported that pCR is not predictive of survival also in luminal B/HER2+ tumors (steroid hormone receptors positive, HER2+) even when neoadjuvant trastuzumab is administered (von Minckwitz et al. 2012). Moreover, there is still no general consensus regarding the definition of pCR, main issues being related to whether or not it should include the presence of noninvasive cancer (von Minckwitz et al. 2010, 2012; Ogston et al. 2003; Chevallier et al. 1993; Sataloff et al. 1995; Green et al. 2005).

The reported high variability of pCR rates in response to neoadjuvant trastuzumab treatment, together with scanty literature data in current clinical practice (Chumsri et al. 2010; Shimizu et al. 2009; Wang et al. 2010; Horiguchi et al. 2011), prompted us to investigate whether the effectiveness of neoadjuvant trastuzumab in association with chemotherapy in ‘real world’ treatment of HER2+ breast cancer patients is comparable to that observed in randomized controlled trials (RCTs).

## Methods

Two hundred and five consecutive patients with early or locally advanced, HER2+, breast cancer, diagnosed in 10 Italian Medical Oncology Units between July 2003 and October 2011, were retrospectively reviewed. All patients were initially candidates for mastectomy and treated by NST. Diagnosis of invasive breast cancer was established by core biopsy of the primary tumor. Patients with bilateral breast cancer, more than one primary tumor and metastatic disease, were excluded. All patients received preoperative trastuzumab in association with chemotherapy. Chemotherapy regimens administered with trastuzumab included different schemes of treatment (Table 1). Hematopoietic growth factors were used according to local practice.

**Table 1:** Neoadjuvant trastuzumab and chemotherapy in 205 patients with operable or locally advanced HER2-positive breast cancer

Regimens	*N* (%)
Trastuzumab concomitant to taxanes	123 (60.0)
After anthra-based regimens	
EC/FEC → Trastuzumab + taxanes	65 (31.7)
EC→ Trastuzumab + docetaxel + carboplatin or capecitabine	35 (17.1)
TEC → Trastuzumab + paclitaxel	2 (0.1)
NPLDoxo + docetaxel→ Trastuzumab + docetaxel	1 (0.5)
Before anthra-based regimens	
Trastuzumab + paclitaxel → EC	2 (0.10)
Nonanthra-based regimens	
Trastuzumab + taxanes	14 (6.9)
MTX + docetaxel → Trastuzumab + docetaxel	3 (1.5)
Trastuzumab + docetaxel + carboplatin	1 (0.5)
Trastuzumab concomitant to anthracyclines and taxanes	77 (37.57)
Trastuzumab + taxanes → Trastuzumab + FEC or EC	67 (32.7)
Trastuzumab + NPLDoxo + CTX→ Trastuzumab + paclitaxel + carboplatin	5 (2.4)
Trastuzumab + FEC or E → Trastuzumab + taxanes	5 (2.4)
Trastuzumab concomitant to other schemes	5 (2.43)

*EC* Epirubicin and cyclophosphamide, *FEC* fluorouracil, epirubicin and cyclophosphamide, *Taxanes* paclitaxel or docetaxel, *TEC* docetaxel, epirubicin and cyclophosphamide, *NPLDoxo* nonpegylated liposomal doxorubicin, *MTX* methotrexate, *CTX* cyclophosphamide, *E* epirubicin

Trastuzumab was continued postoperatively to complete 52 weeks of treatment in 195 patients. Among 125 patients with steroid hormone receptor-positive tumors, 56 patients were treated with adjuvant tamoxifen, 55 patients with aromatase inhibitors (anastrozole or letrozole), 2 patients with tamoxifen followed by exemestane, 2 patients with LHRH analog and 11 patients did not receive any adjuvant hormonal therapy. Surgical procedures consisted of mastectomy or breast-conserving surgery (BCS) and axillary lymph node dissection. Radiotherapy was administered to patients who underwent BCS and to patients who underwent mastectomy, but had initial stage cT3–T4, cN2 or cN3 disease. The study was approved by the independent ethics committees of participating institutions.

### Pathological assessments

Immunohistochemical assessment of HER2, ER, PgR was performed on pretreatment biopsies and surgical specimens by pathologists of participating centers. Neoadjuvant trastuzumab has been administered only to patients whose tumors scored 3+ by HercepTest™ (Dako Italia, Milan, Italy) and/or were FISH, CISH or SISH positive for HER2 gene amplification. Steroid hormone receptors’ status was considered positive if ≥1 % of tumor cells stained for ER and/or PgR. Immunohistochemical detection of Ki-67 was performed using the MIB-1 antibody (Dowsett et al. 2011), and the positivity cutoff value was set at 14 % (Goldhirsch et al. 2011). The nuclear grade was assessed according to the Nottingham grading system (Elston and Ellis 1991). The American Joint Committee on Cancer staging system, 7th ed (2010), was used for tumor staging. Locoregional recurrence (LRR) was defined as any histologically proven chest wall recurrence in those patients who underwent mastectomy, any ipsilateral breast recurrence in those achieving breast conservation and any recurrence in the axillary, supraclavicular or internal mammary nodes.

### Data collection

A retrospective review of clinical, pathological and treatment data for all patients was carried out, and data were entered on an anonymized database. The cutoff date for follow-up was set on October 31, 2012. Since patients enrollment started in 2003, complete data information was not available for all 205 patients; thus, denominators may vary throughout the article. Patients were followed up at 6-month intervals over the first 5 years and at 12-month intervals thereafter.

### Study endpoints and statistics

pCR was defined as either the absence of invasive and noninvasive breast cancer in the breast and axillary lymph nodes (ypT0 and ypN0, later on referred to as pCR/0) or the absence of invasive breast cancer in the breast and axillary lymph nodes (ypT0/is and ypN0, later on referred to as pCR/is), and the latter also classified as Stage 0 (Kuerer et al. 1999; Kaufmann et al. 2010). Disease-free survival (DFS) was calculated from the time of breast surgery to the first occurrence of local relapse, distant metastasis or intercurrent deaths without distant recurrence, and cancer-specific survival (CSS) was calculated from the date of surgery to that of death from breast cancer or last follow-up examination. Two patients were reported to die from causes other than breast cancer. Survivors were censored at the date of last contact. The relationships between patients/tumor characteristics and pCR were assessed by Pearson’s *χ* ^2^ or Fisher’s exact test, as appropriate. Univariate models were developed, and all significant univariate predictors were considered for inclusion in multivariate models. The prediction of pCR was evaluated with a stepwise multivariate logistic regression model. DFS and CSS were estimated using Kaplan–Meier analysis; the log-rank test and Tarone–Ware test for trend were used to assess differences between subgroups (Massarweh et al. 2006; Tarone 1975; Kaplan and Meier 1958). The hazard ratio (HR) and the 95 % confidence intervals (95 % CI) were estimated for each variable. A multivariate Cox proportional hazards model was carried out to assess the relative influence of prognostic factors on survival. Enter and remove limits were *P* = 0.10 and *P* = 0.15, respectively. The assessment of interactions between significant investigation variables was taken into account when developing the multivariate model. All of the tests were two-sided, and *P* ≤ 0.05 was considered significant. All statistical analyses were performed using the SPSS Statistic software 19 (SPSS Inc., Chicago, III).

## Results

### Patients’ characteristics and response to NST

From July 2003 to April 2012, 205 patients with HER2+ early or LABC, candidates for mastectomy, were treated with neoadjuvant trastuzumab in association with different schemes of chemotherapy in 10 Italian Medical Oncology Units. Median age at diagnosis was 48.1 years (range 25–77 years) with 19 (9.3 %) patients being younger than 40 years and 11 (5.4 %) older than 65 years. The associations of baseline clinical characteristics and treatment parameters with pCR/0 and pCR/is are reported in univariate analysis in Table 2. Eighty-seven percent of the tumors (179/205) were invasive ductal carcinomas with a high proliferative activity (62.0 % with Ki-67 ≥14 %). Most tumors (58.0 %) were clinical T_1_–T_2_; tumor grade was 1–2 in 70 (34.2 %) and grade 3 in 74 (36.0 %) tumors. Luminal B/HER2+ (HER2+, ER+ and/or PgR+) molecular subclass was represented in 61.0 % (125/205) of cases and nonluminal/HER2+ subclass in 39.0 % (80/205).

**Table 2:** Association of clinical characteristics and pCR in univariate analysis

	*N* (%)	pCR/0 (*ypT0, ypN0*) *N* (%)	*P* value	pCR/is (*ypT0/is, ypN0*) *N* (%)	*P* value
Histologic type					
Ductal	179 (87.3)	44 (24.6)	0.76	86 (48.0)	0.36
Lobular	12 (5.9)	4 (33.3)		6 (50.0)	
Others	14 (6.8)	3 (21.4)		4 (28.6)	
Clinical T					
T_1_–T_2_	119 (58.0)	34 (28.6)	0.84	61 (51.3)	0.37
T_3_–T_4_	59 (28.8)	16 (27.1)		26 (44.1)	
Unknown^	27 (13.2)				
Grade					
1–2	70 (34.2)	22 (31.4)	0.83	34 (48.6)	0.74
3	74 (36.0)	22 (29.7)		38 (51.3)	
Unknown^	61 (29.8)				
Ki-67					
<14 %	26 (12.7)	3 (11.5)	0.05	12 (46.2)	0.75
≥14 %	127 (62.0)	38 (29.9)		63 (49.6)	
Unknown^	52 (25.4)				
Molecular subclasses					
HER2+, ER+ and/or PgR+°	125 (61.0)	20 (16.0)	0.001	51 (41.1)	0.05
HER2+, ER−, PgR−*	80 (39.0)	30 (37.5)		44 (55.0)	
NST duration					
≤21 weeks	75 (36.6)	14 (18.7)	0.12	35 (46.7)	0.97
>21 weeks	130 (63.4)	37 (28.5)		61 (46.9)	
Trastuzumab duration in NST					
≤12 weeks	104 (50.8)	20 (19.2)	0.06	45 (43.3)	0.30
>12 weeks	101 (49.3)	31 (30.7)		51 (50.5)	

*HER2* Human epidermal growth factor type 2, *ER* estrogen receptors, *PgR* progesteron receptors

^ Unknown were not included in univariate analysis, ° Luminal B/HER2+, * nonluminal/HER2+

Patients were treated with many different chemotherapy regimens. However, 90 % (186/205) of patients received schemes including anthracyclines and all but 3 patients (99 %) received taxanes, either paclitaxel or docetaxel (Table 1). NST was administered for more than 21 weeks (median: 24, range 9–30 weeks) in 130/205 (63.4 %) patients, while trastuzumab was given for more than 12 weeks (median: 12 weeks; range 5–27 weeks) in 101/205 (49.3 %) patients. pCR/0 was obtained in 51/205 (24.8 %) patients and pCR/is in 96/205 (46.8 %) patients. As shown in Table 2, the absence of steroid hormone receptors was highly predictive of pCR/0 (*P* = 0.001), less of pCR/is (*P* = 0.05). There was a trend toward higher rates of pCR/0 for tumors with a high proliferative index (*P* = 0.05) and for a longer duration of neoadjuvant trastuzumab therapy (*P* = 0.06). At multivariate logistic regression, nonluminal/HER2+ tumors (OR 4.03; 95 % CI 1.85–8.74; *P* < 0.0001) and treatment with trastuzumab for more than 12 weeks (OR 2.49; 95 % CI 1.10–5.22; *P* = 0.03) were independent predictors of pCR/0 (Table 3). No other predictive factors were identified either for pCR/0 or pCR/is.

**Table 3:** Predictive factors of pCR/0 according to the multivariate logistic regression model

Variables	pCR/0	
	OR (95% CI)	*P* value
Molecular subclasses		
HER2+, ER−, PgR−* versus HER2+, ER+ and/or PgR+°	4.03 (1.85–8.74)	<0.0001
Trastuzumab duration in NST		
>12 weeks versus ≤12 weeks	2.40 (1.10–5.22)	0.03

*HER2* Human epidermal growth factor type 2, *ER* estrogen receptors, *PgR* progesteron receptors, *OR* odds ratio, *CI* confidence intervals

° Luminal B/HER2+, * nonluminal/HER2+

### Patients’ characteristics and survival

Breast conservative surgery was performed in 97/205 (47.3 %) patients, and radiotherapy was given to 45 of 108 (41.7 %) patients who underwent mastectomy (Table 4). All patients had axillary lymph node dissection. Residual invasive tumors in the breast were detected in 101/205 (49.3 %) patients, residual noninvasive tumor in 45/205 (21.9 %) patients and positive axillary nodes in 53/205 (25.9 %) patients. Pathological stage 0 (ypT0, ypT0/is, ypN0) was assessed in 96/205 (46.8 %), stage I in 35/205 (17.1 %) and stage II–III in 74/205 (36.1 %) patients. Adjuvant chemotherapy was administered to 14/205 (6.8 %) patients and adjuvant hormonal therapy to 114/125 (91.2 %) patients with steroid hormone receptors-positive tumors. In 195/205 (95.1 %) patients, trastuzumab was continued postoperatively to complete 52 weeks of treatment.

**Table 4:** Clinical characteristics of patients and DFS and CSS in univariate analysis

	*N* (%)	5-yr DFS %	*P* value″	5-yr CSS %	*P* value″
Molecular subclasses at diagnosis					
HER2+, ER+ and/or PgR+°	125 (61.0)	78.4	0.06	89.2	0.04
HER2+, ER−, PgR− *	80 (39.0)	66.0	0.05	73.1	0.06
pCR/0 (ypT0, ypN0)					
Yes	51 (24.8)	86.3	0.11	83.5	0.71
No	154 (75.2)	68.7	0.18	82.9	0.55
pCR/is (ypT0/is, ypN0)					
Yes	96 (46.8)	82.3	0.12	86.3	0.18
No	109 (53.2)	65.7	0.12	79.7	0.10
Type of surgery					
Breast conservative surgery	97 (47.3)	77.9	0.08	86.0	0.03
Mastectomy	108 (52.7)	67.9	0.05	76.9	0.01
Radiotherapy					
Yes	142 (69.3)	79.5	0.96	83.6	0.32
No	63 (30.8)	72.0	0.73	83.0	0.26
Mastectomy					
With radiotherapy	45 (41.7)	52.9	0.29	77.5	0.68
Without radiotherapy	63 (58.3)	77.4	0.48	78.7	0.66
Residual tumor size					
ypT0/is	104 (50.7)	79.1	0.04	84.7	0.02
ypT1	55 (26.9)	75.8	0.03	86.6	0.006
ypT2	37 (18.0)	61.2		63.5	
ypT3	9 (4.4)	55.6		65.6	
Node involvement					
ypN0	152 (74.1)	78.0	0.02	86.5	0.05
ypN+	53 (25.9)	58.9	0.02	66.7	0.05
Pathological stage					
0^	96 (46.8)	82.3	0.005	83.5	0.02
I	35 (17.1)	84.4	0.006	100.0	0.02
II	67 (31.7)	54.5		64.3	
III	7 (3.4)	71.4		75.0	
Adjuvant chemotherapy					
Nil	191(93.2)	76.4	0.63	81.3	0.64
Yes	14 (6.8)	43.6	0.85	85.7	0.54
Adjuvant Trastuzumab					
Nil	10 (4.9)	56.2	0.64	87.5	0.91
Up to 52 weeks of treatment	195 (95.1)	74.7	0.64	70.9	0.81
Adjuvant hormonal therapy					
Nil	91 (44.4)	68.1	0.14	75.0	0.12
Yes	114 (55.6)	77.8	0.09	86.9	0.14

*HER2* Human epidermal growth factor type 2, *ER* estrogen receptors, *PgR* progesteron receptors

° Luminal B/HER2+, * nonluminal/HER2+, ^ ypT0/is, ypN0, ″ the first *P* value is determined by log-rank test and the second one by Tarone-Ware test

**Fig. 1:**
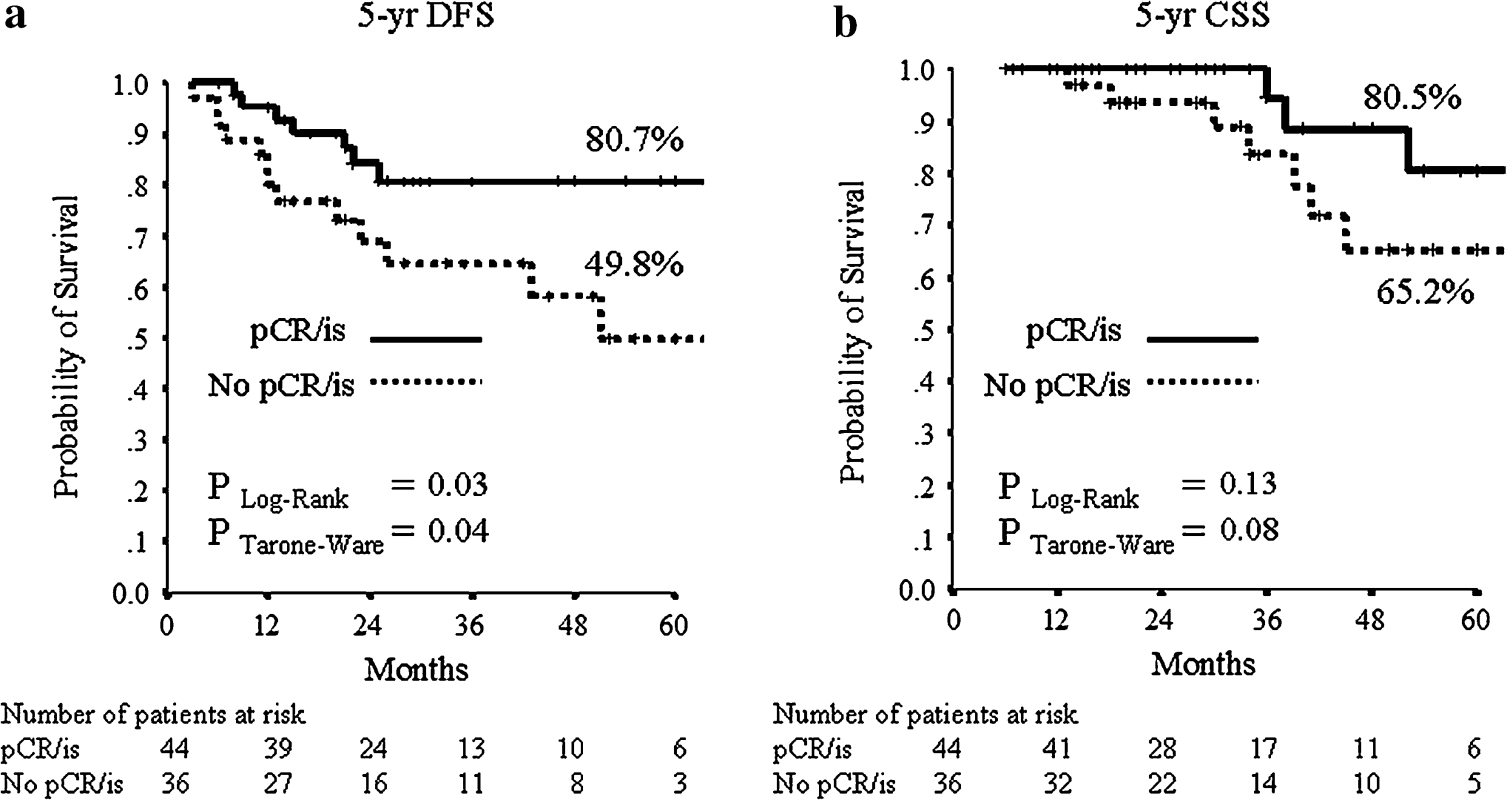
**a** 5-years distant DFS and **b** 5-years CSS stratified by pathological complete response (ypT0/is, ypN0: pCR/is) for the nonluminal/HER2+ subclass, excluding patients with luminal B/HER2+ tumors

**Fig. 2:**
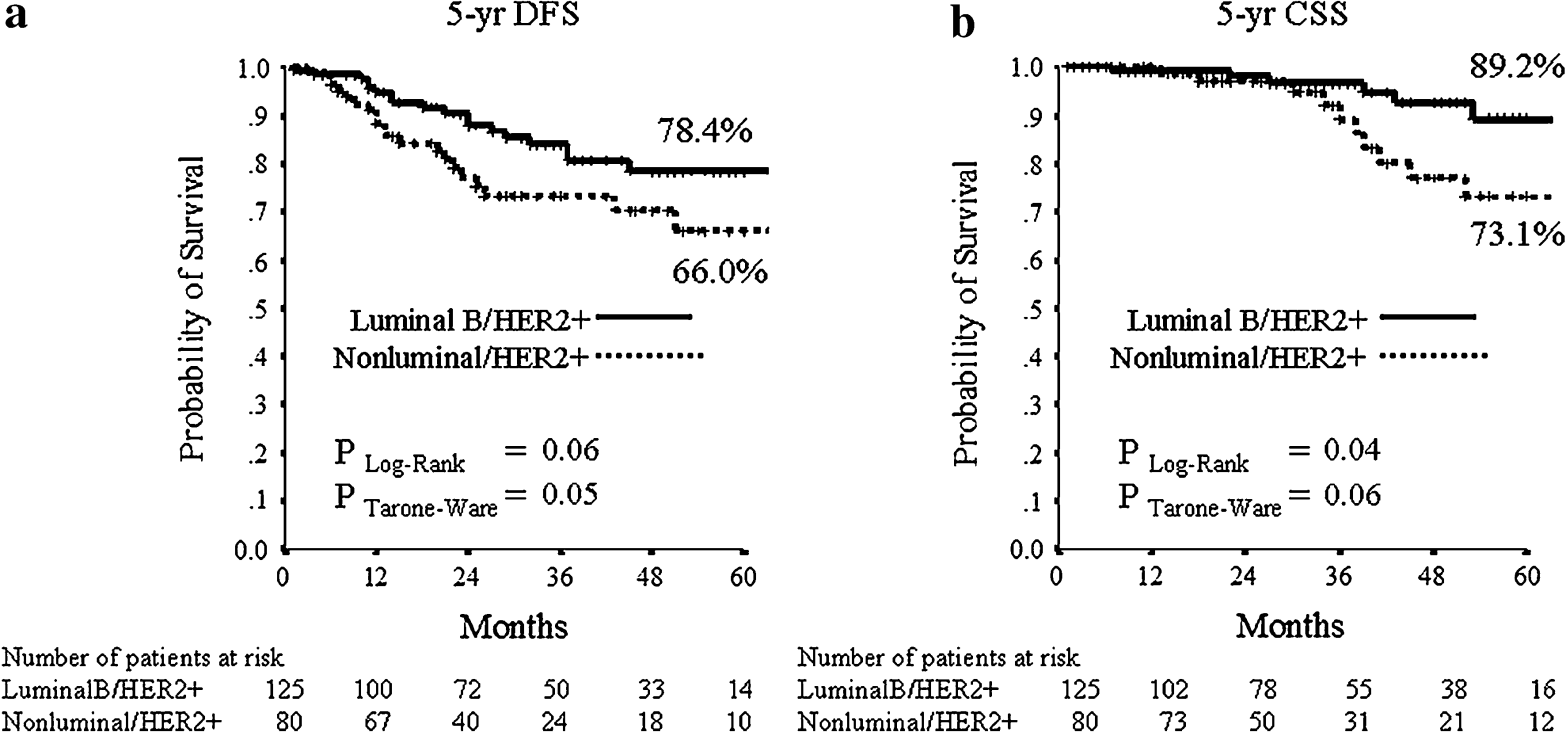
**a** 5-years distant DFS and **b** 5-years CSS stratified by molecular subclass

**Fig. 3:**
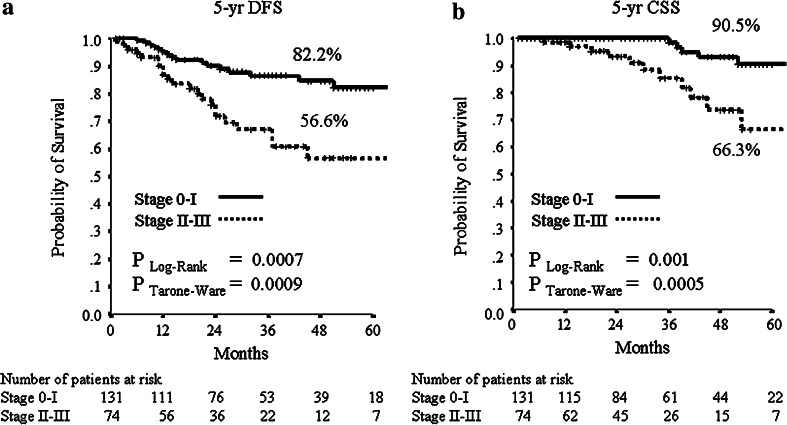
**a** 5-years distant DFS and **b** 5-years CSS stratified pathological stage at surgery

The median follow-up period was 32 months (range 2–106 months). Median DFS and CSS have not been reached at the time of analysis. The mean DFS time was 79 months (95 % CI 70–89), and the mean OS was 94 (95 % CI 89–100) months. During follow-up, 15/205 (7.3 %) patients had local relapse, 30/205 (14.6 %) developed distant metastases (10 of them in the central nervous system) and 18/205 (8.8 %) died from breast cancer. The 5-year estimates were 73.3 % for DFS and 82.8 % for CSS in the all patients’ population. For subsequent survival analyses, we show only data using pCR/is since (1) results were superimposable to those obtained with pCR/0 (data not shown), and (2) pCR/is is included in pathological stage 0 definition (ypT0, ypT0/is, ypN0). At Kaplan–Meier analysis, pCR/is was not found to be a predictive factor for DFS and CSS (data not shown). However, in the molecular subclass of nonluminal/HER2+ (HER2+, ER−, PgR−) pCR/is was predictive of better outcome in terms of DFS (*P* = 0.04; HR = 0.38, 95 % CI 0.15–0.96) with a trend toward significance for CSS (*P* = 0.25; HR = 0.48, 95 % CI 0.14–1.66, Fig. 1). Patients with nonluminal/HER2+ tumors (HER2+, ER−, PgR−) showed a trend toward a worse DFS (*P* = 0.06; HR = 1.82, 95 % CI 0.96–3.45) and CSS (*P* = 0.053; HR = 2.55, 95 % CI 0.99–6.58, Fig. 2) compared to those with luminal B/HER2+. Moreover, patients with pathological stage II–III at surgery were more likely to have a shorter DFS (*P* = 0.001; HR = 2.92, 95 % CI 1.53–5.56) and to die from breast cancer (*P* = 0.01; HR = 3.52, 95 % CI 1.36–9.12, Fig. 3). Type of surgery, residual tumor size, node positivity, pathological stages were also predictive factors of DFS and CSS at univariate analysis (Table 4). At multivariate analysis, the predictive factors of DFS and CSS were the nonluminal/HER2+ molecular subclass (DFS: *P* = 0.01 and CSS: *P* = 0.01) and pathological stage II–III at surgery (DFS: *P* < 0.0001 and CSS: *P* = 0.001) (Table 5).

**Table 5:** Prognostic factors according to the multivariate Cox regression model

Variables	DFS		CSS	
	HR (95% CI)	*P* value	HR (95% CI)	*P* value
Molecular subclasses				
HER2+, ER−, PgR−* versus HER2+, ER+ and/or PgR+°	2.38 (1.24–4.57)	0.01	3.71 (1.32–10.39)	0.01
Pathological stage				
II–III versus 0–I	3.51 (1.81–6.81)	<0.0001	6.36 (2.15–18.74)	0.001

*DFS* Disease-free survival, *CSS*-cancer specific survival, *HER2* human epidermal growth factor type 2, *ER* estrogen receptors, *PgR* progesteron receptors

° Luminal B/HER2+, * nonluminal/HER2+, *HR* hazard ratio, *CI* confidence intervals

## Discussion

Nowadays we assist to an increasing debate on the relative value of RCTs in assisting decision making in day-to-day clinical practice, being this the main endpoint of comparative effectiveness research (Lyman and Levine 2012; Korn and Freidlin 2012). Although RCTs remain the gold standard for comparing treatments, patient selection criteria and the lack of flexibility in protocol-specified dose modifications and toxicity management limit the generalizability of the findings to the individual patient (Hahn and Schilsky 2012). The major questions are whether results of RCTs can be applied to ‘real world’ patients (sometimes with medical co-morbidities or borderline organ function), whether they are also reliably for subsets of patients with different clinical characteristics and whether patients nonenrolled in RCTs have a worst outcome than randomized patients (Korn and Freidlin 2012; Braunholtz et al. 2001; Peppercorn et al. 2004; Hannouf et al. 2012; Engstrom et al. 2012; Tanai et al. 2011). From this point of view, observational retrospective studies are considered alternative good sources of information on the effectiveness of treatments used according to patient and tumor characteristics (Korn and Freidlin 2012; Lyman and Levine 2012).

In the last few years, several phase III RCTs have demonstrated the efficacy of neoadjuvant trastuzumab in association with chemotherapy in HER2+ early or LABC (Gianni et al. 2011; Burstein et al. 2003; Gianni et al. 2010; Untch et al. 2010, 2012; von Minckwitz et al. 2010; Buzdar et al. 2005). A recent meta-analysis of five phase II–III RCTs shows that in the overall population of 515 patients, the absolute pCR rate is 38 % in the trastuzumab arm, with no data on survival (Valachis et al. 2011).

Here we report the effectiveness of neoadjuvant trastuzumab in association with different chemotherapy regimens, in a heterogeneous series of 205 patients with HER2+ operable breast cancer, treated in 10 Italian Medical Oncology Units. Our results show that (1) pCR/0 is obtained in 24.8 % and pCR/is in 46.8 % of the patients; (2) nonluminal/HER2+ subclass and neoadjuvant trastuzumab treatment for more than 12 weeks are predictive factors of pCR/0; (3) pCR (calculated either including or not residual noninvasive cancer cells) is predictive of DFS and CSS only in the nonluminal/HER2+ subclass; and (4) luminal B/HER2+ tumors and pathological stage 0–I disease at surgery are associated with a better DFS and CSS.

Our overall results deserve some considerations. Here we report that neoadjuvant trastuzumab for more than 12 weeks of treatment is associated with a significantly higher rate of pCR/0 than shorter treatments (OR 2.49; 95 % CI 1.10–5.22; *P* = 0.03). To our knowledge, no RCTs evaluated clinical effectiveness of longer versus shorter neoadjuvant trastuzumab administration, and this issue is object of debate (Untch et al. 2011). A pooled analysis of the German neoadjuvant chemotherapy trials found little evidence that an increasing number of trastuzumab cycles is associated with higher pCR rates in HER2+ patients (von Minckwitz et al. 2011). A retrospective study on neoadjuvant therapy for HER2+ breast cancer reported that patients treated with a regimen in which trastuzumab was administered concurrently with anthracyclines for 24 weeks (trastuzumab and paclitaxel for 12 weeks followed by trastuzumab and FEC for 12 weeks) achieved a higher pCR rate compared to that observed in patients treated with 18 weeks of trastuzumab in association with a nonanthracycline-based regimen, docetaxel and carboplatin (60.6 vs 43.3 %; *P* = 0.016) (Bayraktar et al. 2012). Authors linked the higher pCR rate to the concurrent trastuzumab administration with a sequential regimen of a taxane followed by an anthracycline-containing regimen and not to the longer trastuzumab exposure (Bayraktar et al. 2012). However, in our cohort, over 90 % of patients received schemes of chemotherapy including anthracyclines and almost all received taxanes, though with different sequences and combinations.

The pCR/0 (24.8 %) and pCR/is (46.8 %) rate observed in our series is within the range of the pCR rates reported in the literature (Untch et al. 2012; Valachis et al. 2011; Ismael et al. 2012). Similarly to von Minckwitz and coll. (von Minckwitz et al. 2012), we had a higher rate of pCR/0 and pCR/is in patients with the worst prognostic factors, for example high proliferative activity and nonluminal/HER2+ tumors. Moreover, pCR/0 and pCR/is achieved similar results in terms of DFS and CSS, being both predictive of better outcome only in the molecular subclass of luminal B/HER2+ tumors.

The long-term outcome of patients included in this analysis was significantly affected by steroid hormone receptors expression and residual tumor burden at surgery. Luminal B/HER2+ molecular subclass was associated with a better outcome, as reported for luminal A, luminal B/HER2-negative and luminal B/HER2+ tumors in other series (Goldhirsch et al. 2011; Angelucci et al. 2012; Houssami et al. 2012; Tran and Bedard 2011). Pathological stage 0–I was also predictive of better outcome in our cohort of patients, in line with data showing that among patients with residual disease, a higher tumor burden at surgery is predictive of a worst outcome (Fisher et al. 1998; Angelucci et al. 2012; Cance et al. 2002; Carey et al. 2005).

The strength of this multicenter retrospective observational study is that it represents data from a cohort of ‘real world’ individual patients with a wide age range (from 25 to 77 years), treated with neoadjuvant trastuzumab outside of clinical trials in association with different schemes of chemotherapy regimens (Table 1), with different duration of NST (from 9 to 30 weeks), and trastuzumab treatment (from 5 to 27 weeks), in 10 different oncology units with optimal follow-up adherence (no patient lost to follow-up). The main weaknesses are represented (1) by the fact that inclusion/exclusion criteria, therapeutic regimens, dose modifications and toxicity management for neoadjuvant trastuzumab treatment were not defined from a predefined study protocol, thus leaving them to the single oncology unit decision and (2) preoperative complete data information, such as clinical T, grade and Ki-67, were not available for all 205 patients,

In conclusion, this study shows that a longer duration of trastuzumab treatment and the absence of estrogen and/or progesterone receptors are significantly associated with a higher rate of pCR/0. pCR/0 and pCR/is are predictive of a better outcome only in luminal B/HER2+ tumors. High residual tumor burden after NST (i.e., pathological stage II–III at surgery) and the absence of estrogen and/or progesterone receptors are both independent predictors of shorter DFS and CSS.

## References

[CR1] Angelucci D, Tinari N, Grassadonia A, Cianchetti E, Ausili-Cefaro G, Iezzi L, Zilli M, Grossi S, Ursini LA, Scognamiglio MT, Castrilli G, De Tursi M, Noccioli P, Cioffi P, Iacobelli S, Natoli C (2012) Long-term outcome of neoadjuvant systemic therapy for locally advanced breast cancer in routine clinical practice. J Cancer Res Clin Oncol. doi:10.1007/s00432-012-1325-9 23052698 10.1007/s00432-012-1325-9 PMC3549406

[CR2] Anton A, Ruiz A, Plazaola A, Calvo L, Segui MA, Santaballa A, Munoz M, Sanchez P, Miguel A, Carrasco E, Lao J, Camps J, Alfaro J, Antolin S, Camara MC (2011) Phase II clinical trial of liposomal-encapsulated doxorubicin citrate and docetaxel, associated with trastuzumab, as neoadjuvant treatment in stages II and IIIA HER2-overexpressing breast cancer patients. GEICAM 2003–03 study. Ann Oncol 22(1):74–79. doi:10.1093/annonc/mdq317 20603435 10.1093/annonc/mdq317

[CR3] Bayraktar S, Gonzalez-Angulo AM, Lei X, Buzdar AU, Valero V, Melhem-Bertrandt A, Kuerer HM, Hortobagyi GN, Sahin AA, Meric-Bernstam F (2012) Efficacy of neoadjuvant therapy with trastuzumab concurrent with anthracycline- and nonanthracycline-based regimens for HER2-positive breast cancer. Cancer 118(9):2385–2393. doi:10.1002/cncr.26555 21953213 10.1002/cncr.26555 PMC3274632

[CR4] Bear HD, Anderson S, Smith RE, Geyer CE Jr, Mamounas EP, Fisher B, Brown AM, Robidoux A, Margolese R, Kahlenberg MS, Paik S, Soran A, Wickerham DL, Wolmark N (2006) Sequential preoperative or postoperative docetaxel added to preoperative doxorubicin plus cyclophosphamide for operable breast cancer: National Surgical Adjuvant Breast and Bowel Project Protocol B-27. J Clin Oncol 24(13):2019–2027. doi:10.1200/jco.2005.04.1665 16606972 10.1200/JCO.2005.04.1665

[CR5] Braunholtz DA, Edwards SJ, Lilford RJ (2001) Are randomized clinical trials good for us (in the short term)? Evidence for a “trial effect”. J Clin Epidemiol 54(3):217–224 11223318 10.1016/s0895-4356(00)00305-x

[CR6] Brufsky A (2010) Trastuzumab-based therapy for patients with HER2-positive breast cancer: from early scientific development to foundation of care. Am J Clin Oncol 33(2):186–195. doi:10.1097/COC.0b013e318191bfb0 19675448 10.1097/COC.0b013e318191bfb0

[CR7] Burstein HJ, Harris LN, Gelman R, Lester SC, Nunes RA, Kaelin CM, Parker LM, Ellisen LW, Kuter I, Gadd MA, Christian RL, Kennedy PR, Borges VF, Bunnell CA, Younger J, Smith BL, Winer EP (2003) Preoperative therapy with trastuzumab and paclitaxel followed by sequential adjuvant doxorubicin/cyclophosphamide for HER2 overexpressing stage II or III breast cancer: a pilot study. J Clin Oncol 21(1):46–53 12506169 10.1200/JCO.2003.03.124

[CR8] Buzdar AU, Ibrahim NK, Francis D, Booser DJ, Thomas ES, Theriault RL, Pusztai L, Green MC, Arun BK, Giordano SH, Cristofanilli M, Frye DK, Smith TL, Hunt KK, Singletary SE, Sahin AA, Ewer MS, Buchholz TA, Berry D, Hortobagyi GN (2005) Significantly higher pathologic complete remission rate after neoadjuvant therapy with trastuzumab, paclitaxel, and epirubicin chemotherapy: results of a randomized trial in human epidermal growth factor receptor 2-positive operable breast cancer. J Clin Oncol 23(16):3676–3685. doi:10.1200/jco.2005.07.032 15738535 10.1200/JCO.2005.07.032

[CR9] Buzdar AU, Valero V, Ibrahim NK, Francis D, Broglio KR, Theriault RL, Pusztai L, Green MC, Singletary SE, Hunt KK, Sahin AA, Esteva F, Symmans WF, Ewer MS, Buchholz TA, Hortobagyi GN (2007) Neoadjuvant therapy with paclitaxel followed by 5-fluorouracil, epirubicin, and cyclophosphamide chemotherapy and concurrent trastuzumab in human epidermal growth factor receptor 2-positive operable breast cancer: an update of the initial randomized study population and data of additional patients treated with the same regimen. Clin Cancer Res 13(1):228–233. doi:10.1158/1078-0432.ccr-06-1345 17200359 10.1158/1078-0432.CCR-06-1345

[CR10] Cance WG, Carey LA, Calvo BF, Sartor C, Sawyer L, Moore DT, Rosenman J, Ollila DW, Graham M 2nd (2002) Long-term outcome of neoadjuvant therapy for locally advanced breast carcinoma: effective clinical downstaging allows breast preservation and predicts outstanding local control and survival. Ann Surg 236(3):295–302. doi:10.1097/01.sla.0000027526.67560.64 12192316 10.1097/01.SLA.0000027526.67560.64 PMC1422583

[CR11] Carey LA, Metzger R, Dees EC, Collichio F, Sartor CI, Ollila DW, Klauber-DeMore N, Halle J, Sawyer L, Moore DT, Graham ML (2005) American joint committee on cancer tumor-node-metastasis stage after neoadjuvant chemotherapy and breast cancer outcome. J Natl Cancer Inst 97(15):1137–1142. doi:10.1093/jnci/dji206 16077072 10.1093/jnci/dji206

[CR12] Chevallier B, Roche H, Olivier JP, Chollet P, Hurteloup P (1993) Inflammatory breast cancer. Pilot study of intensive induction chemotherapy (FEC-HD) results in a high histologic response rate. Am J Clin Oncol 16(3):223–228 8338056

[CR13] Chumsri S, Jeter S, Jacobs LK, Nassar H, Armstrong DK, Emens LA, Fetting JH, Lange JR, Riley C, Tsangaris TN, Wolff AC, Zellars R, Zhang Z, Stearns V (2010) Pathologic complete response to preoperative sequential doxorubicin/cyclophosphamide and single-agent taxane with or without trastuzumab in stage II/III HER2-positive breast cancer. Clin Breast Cancer 10(1):40–45. doi:10.3816/CBC.2010.n.005 20133257 10.3816/CBC.2010.n.005

[CR14] Colleoni M, Bagnardi V, Rotmensz N, Gelber RD, Viale G, Pruneri G, Veronesi P, Torrisi R, Cardillo A, Montagna E, Campagnoli E, Luini A, Intra M, Galimberti V, Scarano E, Peruzzotti G, Goldhirsch A (2009) Increasing steroid hormone receptors expression defines breast cancer subtypes non responsive to preoperative chemotherapy. Breast Cancer Res Treat 116(2):359–369. doi:10.1007/s10549-008-0223-y 18941889 10.1007/s10549-008-0223-y

[CR15] Costa RB, Kurra G, Greenberg L, Geyer CE (2010) Efficacy and cardiac safety of adjuvant trastuzumab-based chemotherapy regimens for HER2-positive early breast cancer. Ann Oncol 21(11):2153–2160. doi:10.1093/annonc/mdq096 20351072 10.1093/annonc/mdq096

[CR16] Coudert BP, Largillier R, Arnould L, Chollet P, Campone M, Coeffic D, Priou F, Gligorov J, Martin X, Trillet-Lenoir V, Weber B, Bleuse JP, Vasseur B, Serin D, Namer M (2007) Multicenter phase II trial of neoadjuvant therapy with trastuzumab, docetaxel, and carboplatin for human epidermal growth factor receptor-2-overexpressing stage II or III breast cancer: results of the GETN(A)-1 trial. J Clin Oncol 25(19):2678–2684. doi:10.1200/jco.2006.09.9994 17515572 10.1200/JCO.2006.09.9994

[CR17] Dawood S, Gonzalez-Angulo AM, Peintinger F, Broglio K, Symmans WF, Kau SW, Islam R, Hortobagyi GN, Buzdar AU (2007) Efficacy and safety of neoadjuvant trastuzumab combined with paclitaxel and epirubicin: a retrospective review of the M. D. Anderson experience. Cancer 110(6):1195–1200. doi:10.1002/cncr.22895 17647266 10.1002/cncr.22895

[CR18] Dowsett M, Nielsen TO, A’Hern R, Bartlett J, Coombes RC, Cuzick J, Ellis M, Henry NL, Hugh JC, Lively T, McShane L, Paik S, Penault-Llorca F, Prudkin L, Regan M, Salter J, Sotiriou C, Smith IE, Viale G, Zujewski JA, Hayes DF (2011) Assessment of Ki67 in breast cancer: recommendations from the International Ki67 in Breast Cancer working group. J Natl Cancer Inst 103(22):1656–1664. doi:10.1093/jnci/djr393 21960707 10.1093/jnci/djr393 PMC3216967

[CR19] Elston CW, Ellis IO (1991) Pathological prognostic factors in breast cancer. I. The value of histological grade in breast cancer: experience from a large study with long-term follow-up. Histopathology 19(5):403–410 1757079 10.1111/j.1365-2559.1991.tb00229.x

[CR20] Engstrom C, Jamieson GG, Devitt PG, Irvine T, Watson DI (2012) Impact of participation in randomized trials on outcome following surgery for gastro-oesophageal reflux. Br J Surg 99(3):381–386. doi:10.1002/bjs.8666 22231692 10.1002/bjs.8666

[CR21] Fisher B, Brown A, Mamounas E, Wieand S, Robidoux A, Margolese RG, Cruz AB Jr, Fisher ER, Wickerham DL, Wolmark N, DeCillis A, Hoehn JL, Lees AW, Dimitrov NV (1997) Effect of preoperative chemotherapy on local-regional disease in women with operable breast cancer: findings from National Surgical Adjuvant Breast and Bowel Project B-18. J Clin Oncol 15(7):2483–2493 9215816 10.1200/JCO.1997.15.7.2483

[CR22] Fisher B, Bryant J, Wolmark N, Mamounas E, Brown A, Fisher ER, Wickerham DL, Begovic M, DeCillis A, Robidoux A, Margolese RG, Cruz AB Jr, Hoehn JL, Lees AW, Dimitrov NV, Bear HD (1998) Effect of preoperative chemotherapy on the outcome of women with operable breast cancer. J Clin Oncol 16(8):2672–2685 9704717 10.1200/JCO.1998.16.8.2672

[CR23] Garnock-Jones KP, Keating GM, Scott LJ (2010) Trastuzumab: a review of its use as adjuvant treatment in human epidermal growth factor receptor 2 (HER2)-positive early breast cancer. Drugs 70(2):215–239. doi:10.2165/11203700-000000000-00000 20108993 10.2165/11203700-000000000-00000

[CR24] Gianni L, Eiermann W, Semiglazov V, Manikhas A, Lluch A, Tjulandin S, Zambetti M, Vazquez F, Byakhow M, Lichinitser M, Climent MA, Ciruelos E, Ojeda B, Mansutti M, Bozhok A, Baronio R, Feyereislova A, Barton C, Valagussa P, Baselga J (2010) Neoadjuvant chemotherapy with trastuzumab followed by adjuvant trastuzumab versus neoadjuvant chemotherapy alone, in patients with HER2-positive locally advanced breast cancer (the NOAH trial): a randomised controlled superiority trial with a parallel HER2-negative cohort. Lancet 375(9712):377–384. doi:10.1016/s0140-6736(09)61964-4 20113825 10.1016/S0140-6736(09)61964-4

[CR25] Gianni L, Dafni U, Gelber RD, Azambuja E, Muehlbauer S, Goldhirsch A, Untch M, Smith I, Baselga J, Jackisch C, Cameron D, Mano M, Pedrini JL, Veronesi A, Mendiola C, Pluzanska A, Semiglazov V, Vrdoljak E, Eckart MJ, Shen Z, Skiadopoulos G, Procter M, Pritchard KI, Piccart-Gebhart MJ, Bell R (2011) Treatment with trastuzumab for 1 year after adjuvant chemotherapy in patients with HER2-positive early breast cancer: a 4-year follow-up of a randomised controlled trial. Lancet Oncol 12(3):236–244. doi:10.1016/s1470-2045(11)70033-x 21354370 10.1016/S1470-2045(11)70033-X

[CR26] Goldhirsch A, Wood WC, Coates AS, Gelber RD, Thurlimann B, Senn HJ (2011) Strategies for subtypes–dealing with the diversity of breast cancer: highlights of the St. Gallen International Expert Consensus on the Primary Therapy of Early Breast Cancer 2011. Ann Oncol 22(8):1736–1747. doi:10.1093/annonc/mdr304 21709140 10.1093/annonc/mdr304 PMC3144634

[CR27] Green MC, Buzdar AU, Smith T, Ibrahim NK, Valero V, Rosales MF, Cristofanilli M, Booser DJ, Pusztai L, Rivera E, Theriault RL, Carter C, Frye D, Hunt KK, Symmans WF, Strom EA, Sahin AA, Sikov W, Hortobagyi GN (2005) Weekly paclitaxel improves pathologic complete remission in operable breast cancer when compared with paclitaxel once every 3 weeks. J Clin Oncol 23(25):5983–5992. doi:10.1200/jco.2005.06.232 16087943 10.1200/JCO.2005.06.232

[CR28] Hahn OM, Schilsky RL (2012) Randomized controlled trials and comparative effectiveness research. J Clin Oncol. doi:10.1200/jco.2012.42.2352 10.1200/JCO.2012.42.2352 23071239

[CR29] Hannouf MB, Brackstone M, Xie B, Zaric GS (2012) Evaluating the efficacy of current clinical practice of adjuvant chemotherapy in postmenopausal women with early-stage, estrogen or progesterone receptor-positive, one-to-three positive axillary lymph node, breast cancer. Curr Oncol 19(5):e319–e328. doi:10.3747/co.19.1038 23144580 10.3747/co.19.1038 PMC3457883

[CR30] Harris LN, You F, Schnitt SJ, Witkiewicz A, Lu X, Sgroi D, Ryan PD, Come SE, Burstein HJ, Lesnikoski BA, Kamma M, Friedman PN, Gelman R, Iglehart JD, Winer EP (2007) Predictors of resistance to preoperative trastuzumab and vinorelbine for HER2-positive early breast cancer. Clin Cancer Res 13(4):1198–1207. doi:10.1158/1078-0432.ccr-06-1304 17317830 10.1158/1078-0432.CCR-06-1304

[CR31] Horiguchi J, Oyama T, Koibuchi Y, Yokoe T, Takata D, Ikeda F, Nagaoka H, Rokutanda N, Nagaoka R, Ishikawa Y, Odawara H, Kikuchi M, Sato A, Iino Y, Takeyoshi I (2009) Neoadjuvant weekly paclitaxel with and without trastuzumab in locally advanced or metastatic breast cancer. Anticancer Res 29(2):517–524 19331197

[CR32] Horiguchi J, Oyama T, Takata D, Rokutanda N, Nagaoka R, Odawara H, Tokiniwa H, Tozuka K, Kikuchi M, Sato A, Takeyoshi I (2011) Pathological complete response and prognosis in patients receiving neoadjuvant paclitaxel and trastuzumab with and without anthracyclines for stage II and III, HER2-positive operable breast cancer: a single-institute experience. Anticancer Res 31(9):3041–3046 21868556

[CR33] Houssami N, Macaskill P, von Minckwitz G, Marinovich ML, Mamounas E (2012) Meta-analysis of the association of breast cancer subtype and pathologic complete response to neoadjuvant chemotherapy. Eur J Cancer. doi:10.1016/j.ejca.2012.05.023 22766518 10.1016/j.ejca.2012.05.023

[CR34] Hudis CA (2007) Trastuzumab—mechanism of action and use in clinical practice. N Engl J Med 357(1):39–51. doi:10.1056/NEJMra043186 17611206 10.1056/NEJMra043186

[CR35] Huober J, von Minckwitz G, Denkert C, Tesch H, Weiss E, Zahm DM, Belau A, Khandan F, Hauschild M, Thomssen C, Hogel B, Darb-Esfahani S, Mehta K, Loibl S (2010) Effect of neoadjuvant anthracycline-taxane-based chemotherapy in different biological breast cancer phenotypes: overall results from the GeparTrio study. Breast Cancer Res Treat 124(1):133–140. doi:10.1007/s10549-010-1103-9 20697801 10.1007/s10549-010-1103-9

[CR36] Hurley J, Doliny P, Reis I, Silva O, Gomez-Fernandez C, Velez P, Pauletti G, Powell JE, Pegram MD, Slamon DJ (2006) Docetaxel, cisplatin, and trastuzumab as primary systemic therapy for human epidermal growth factor receptor 2-positive locally advanced breast cancer. J Clin Oncol 24(12):1831–1838. doi:10.1200/jco.2005.02.8886 16549824 10.1200/JCO.2005.02.8886

[CR37] Ismael G, Hegg R, Muehlbauer S, Heinzmann D, Lum B, Kim SB, Pienkowski T, Lichinitser M, Semiglazov V, Melichar B, Jackisch C (2012) Subcutaneous versus intravenous administration of (neo)adjuvant trastuzumab in patients with HER2-positive, clinical stage I–III breast cancer (HannaH study): a phase 3, open-label, multicentre, randomised trial. Lancet Oncol 13(9):869–878. doi:10.1016/s1470-2045(12)70329-7 22884505 10.1016/S1470-2045(12)70329-7

[CR38] Kaplan E, Meier P (1958) Nonparametric estimation from incomplete observations. J Am Stat Assoc 53:457–481

[CR39] Kaufmann M, Hortobagyi GN, Goldhirsch A, Scholl S, Makris A, Valagussa P, Blohmer JU, Eiermann W, Jackesz R, Jonat W, Lebeau A, Loibl S, Miller W, Seeber S, Semiglazov V, Smith R, Souchon R, Stearns V, Untch M, von Minckwitz G (2006) Recommendations from an international expert panel on the use of neoadjuvant (primary) systemic treatment of operable breast cancer: an update. J Clin Oncol 24(12):1940–1949. doi:10.1200/jco.2005.02.6187 16622270 10.1200/JCO.2005.02.6187

[CR40] Kaufmann M, Morrow M, von Minckwitz G, Harris JR (2010) Locoregional treatment of primary breast cancer: consensus recommendations from an international expert panel. Cancer 116(5):1184–1191. doi:10.1002/cncr.24874 20087962 10.1002/cncr.24874

[CR41] Kim SI, Sohn J, Koo JS, Park SH, Park HS, Park BW (2010) Molecular subtypes and tumor response to neoadjuvant chemotherapy in patients with locally advanced breast cancer. Oncology 79(5–6):324–330. doi:10.1159/000322192 21430399 10.1159/000322192

[CR42] Korn EL, Freidlin B (2012) Methodology for comparative effectiveness research: potential and limitations. J Clin Oncol. doi:10.1200/jco.2012.44.8233 10.1200/JCO.2012.44.8233 23071226

[CR43] Kuerer HM, Newman LA, Smith TL, Ames FC, Hunt KK, Dhingra K, Theriault RL, Singh G, Binkley SM, Sneige N, Buchholz TA, Ross MI, McNeese MD, Buzdar AU, Hortobagyi GN, Singletary SE (1999) Clinical course of breast cancer patients with complete pathologic primary tumor and axillary lymph node response to doxorubicin-based neoadjuvant chemotherapy. J Clin Oncol 17(2):460–469 10080586 10.1200/JCO.1999.17.2.460

[CR44] Lyman GH, Levine M (2012) Comparative effectiveness research in oncology: an overview. J Clin Oncol. doi:10.1200/jco.2012.45.9792 10.1200/JCO.2012.45.9792 23071249

[CR45] Mariani G, Fasolo A, De Benedictis E, Gianni L (2009) Trastuzumab as adjuvant systemic therapy for HER2-positive breast cancer. Nat Clin Pract Oncol 6(2):93–104. doi:10.1038/ncponc1298 19107109 10.1038/ncponc1298

[CR46] Massarweh S, Osborne CK, Jiang S, Wakeling AE, Rimawi M, Mohsin SK, Hilsenbeck S, Schiff R (2006) Mechanisms of tumor regression and resistance to estrogen deprivation and fulvestrant in a model of estrogen receptor-positive, HER-2/neu-positive breast cancer. Cancer Res 66(16):8266–8273. doi:10.1158/0008-5472.can-05-4045 16912207 10.1158/0008-5472.CAN-05-4045

[CR47] Mauri D, Pavlidis N, Ioannidis JP (2005) Neoadjuvant versus adjuvant systemic treatment in breast cancer: a meta-analysis. J Natl Cancer Inst 97(3):188–194. doi:10.1093/jnci/dji021 15687361 10.1093/jnci/dji021

[CR48] Mazouni C, Peintinger F, Wan-Kau S, Andre F, Gonzalez-Angulo AM, Symmans WF, Meric-Bernstam F, Valero V, Hortobagyi GN, Pusztai L (2007) Residual ductal carcinoma in situ in patients with complete eradication of invasive breast cancer after neoadjuvant chemotherapy does not adversely affect patient outcome. J Clin Oncol 25(19):2650–2655. doi:10.1200/jco.2006.08.2271 17602071 10.1200/JCO.2006.08.2271

[CR49] Ogston KN, Miller ID, Payne S, Hutcheon AW, Sarkar TK, Smith I, Schofield A, Heys SD (2003) A new histological grading system to assess response of breast cancers to primary chemotherapy: prognostic significance and survival. Breast 12(5):320–327 14659147 10.1016/s0960-9776(03)00106-1

[CR50] Penault-Llorca F, Abrial C, Mouret-Reynier MA, Raoelfils I, Durando X, Leheurteur M, Gimbergues P, Tortochaux J, Cure H, Chollet P (2007) Achieving higher pathological complete response rates in HER-2-positive patients with induction chemotherapy without trastuzumab in operable breast cancer. Oncologist 12(4):390–396. doi:10.1634/theoncologist.12-4-390 17470681 10.1634/theoncologist.12-4-390

[CR51] Peppercorn JM, Weeks JC, Cook EF, Joffe S (2004) Comparison of outcomes in cancer patients treated within and outside clinical trials: conceptual framework and structured review. Lancet 363(9405):263–270. doi:10.1016/s0140-6736(03)15383-4 14751698 10.1016/S0140-6736(03)15383-4

[CR52] Petrelli F, Borgonovo K, Cabiddu M, Ghilardi M, Barni S (2011) Neoadjuvant chemotherapy and concomitant trastuzumab in breast cancer: a pooled analysis of two randomized trials. Anticancer Drugs 22(2):128–135 21218604 10.1097/cad.0b013e32834120aa

[CR53] Pierga JY, Delaloge S, Espie M, Brain E, Sigal-Zafrani B, Mathieu MC, Bertheau P, Guinebretiere JM, Spielmann M, Savignoni A, Marty M (2010) A multicenter randomized phase II study of sequential epirubicin/cyclophosphamide followed by docetaxel with or without celecoxib or trastuzumab according to HER2 status, as primary chemotherapy for localized invasive breast cancer patients. Breast Cancer Res Treat 122(2):429–437. doi:10.1007/s10549-010-0939-3 20480225 10.1007/s10549-010-0939-3

[CR54] Precht LM, Lowe KA, Atwood M, Beatty JD (2010) Neoadjuvant chemotherapy of breast cancer: tumor markers as predictors of pathologic response, recurrence, and survival. Breast J 16(4):362–368. doi:10.1111/j.1524-4741.2010.00935.x 20443786 10.1111/j.1524-4741.2010.00935.x

[CR55] Robidoux A, Buzdar AU, Quinaux E, Jacobs S, Rastogi P, Fourchotte V, Younan RJ, Pajon ER, Shalaby IA, Desai AM, Fehrenbacher L, Geyer CE Jr, Mamounas EP, Wolmark N (2010) A phase II neoadjuvant trial of sequential nanoparticle albumin-bound paclitaxel followed by 5-fluorouracil/epirubicin/cyclophosphamide in locally advanced breast cancer. Clin Breast Cancer 10(1):81–86. doi:10.3816/CBC.2010.n.011 20133263 10.3816/CBC.2010.n.011

[CR56] Ross JS, Slodkowska EA, Symmans WF, Pusztai L, Ravdin PM, Hortobagyi GN (2009) The HER-2 receptor and breast cancer: ten years of targeted anti-HER-2 therapy and personalized medicine. Oncologist 14(4):320–368. doi:10.1634/theoncologist.2008-0230 19346299 10.1634/theoncologist.2008-0230

[CR57] Ruiz M, Salvador J, Bayo J, Lomas M, Moreno A, Valero M, Bernabe R, Vicente D, Jimenez J, Lopez-Ladron A (2008) Phase-II study of weekly schedule of trastuzumab, paclitaxel, and carboplatin followed by a week off every 28 days for HER2+ metastatic breast cancer. Cancer Chemother Pharmacol 62(6):1085–1090. doi:10.1007/s00280-008-0709-7 18365200 10.1007/s00280-008-0709-7

[CR58] Sanchez-Munoz A, Duenas-Garcia R, Jaen-Morago A, Carrasco E, Chacon I, Garcia-Tapiador AM, Ortega-Granados AL, Martinez-Ortega E, Ribelles N, Fernandez-Navarro M, de la Torre-Cabrera C, Duenas B, Rueda AI, Martinez J, Tortosa CR, Martin-Salvago MD, Sanchez-Rovira P (2010) Is it possible to increase pCR in the neoadjuvant treatment with a dose-dense/sequential combination? Results from a phase II Trial combining epirubicin and cyclophosphamide followed by paclitaxel and gemcitabine±trastuzumab in stage II and III breast cancer patients. Am J Clin Oncol 33(5):432–437. doi:10.1097/COC.0b013e3181b4eff9 19952716 10.1097/COC.0b013e3181b4eff9

[CR59] Sataloff DM, Mason BA, Prestipino AJ, Seinige UL, Lieber CP, Baloch Z (1995) Pathologic response to induction chemotherapy in locally advanced carcinoma of the breast: a determinant of outcome. J Am Coll Surg 180(3):297–306 7874340

[CR60] Schott AF, Hayes DF (2012) Defining the benefits of neoadjuvant chemotherapy for breast cancer. J Clin Oncol 30(15):1747–1749. doi:10.1200/jco.2011.41.3161 22508810 10.1200/JCO.2011.41.3161

[CR61] Semiglazov V, Eiermann W, Zambetti M, Manikhas A, Bozhok A, Lluch A, Tjulandin S, Sabadell MD, Caballero A, Valagussa P, Baselga J, Gianni L (2011) Surgery following neoadjuvant therapy in patients with HER2-positive locally advanced or inflammatory breast cancer participating in the NeOAdjuvant Herceptin (NOAH) study. Eur J Surg Oncol 37(10):856–863. doi:10.1016/j.ejso.2011.07.003 21843921 10.1016/j.ejso.2011.07.003

[CR62] Shimizu C, Masuda N, Yoshimura K, Tsuda H, Mano M, Ando M, Tamura K, Fujiwara Y (2009) Long-term outcome and pattern of relapse after neoadjuvant chemotherapy in patients with human epidermal growth factor receptor 2-positive primary breast cancer. Jpn J Clin Oncol 39(8):484–490. doi:10.1093/jjco/hyp052 19477897 10.1093/jjco/hyp052

[CR63] Sikov WM, Dizon DS, Strenger R, Legare RD, Theall KP, Graves TA, Gass JS, Kennedy TA, Fenton MA (2009) Frequent pathologic complete responses in aggressive stages II to III breast cancers with every-4-week carboplatin and weekly paclitaxel with or without trastuzumab: a Brown University Oncology Group Study. J Clin Oncol 27(28):4693–4700. doi:10.1200/jco.2008.21.4163 19720916 10.1200/JCO.2008.21.4163

[CR64] Straver ME, Rutgers EJ, Rodenhuis S, Linn SC, Loo CE, Wesseling J, Russell NS, Oldenburg HS, Antonini N, Vrancken Peeters MT (2010) The relevance of breast cancer subtypes in the outcome of neoadjuvant chemotherapy. Ann Surg Oncol 17(9):2411–2418. doi:10.1245/s10434-010-1008-1 20373039 10.1245/s10434-010-1008-1 PMC2924493

[CR65] Symmans WF, Peintinger F, Hatzis C, Rajan R, Kuerer H, Valero V, Assad L, Poniecka A, Hennessy B, Green M, Buzdar AU, Singletary SE, Hortobagyi GN, Pusztai L (2007) Measurement of residual breast cancer burden to predict survival after neoadjuvant chemotherapy. J Clin Oncol 25(28):4414–4422. doi:10.1200/jco.2007.10.6823 17785706 10.1200/JCO.2007.10.6823

[CR66] Tanai C, Nakajima TE, Nagashima K, Kato K, Hamaguchi T, Yamada Y, Muro K, Shirao K, Kunitoh H, Matsumura Y, Yamamoto S, Shimada Y (2011) Characteristics and outcomes of patients with advanced gastric cancer who declined to participate in a randomized clinical chemotherapy trial. J Oncol Pract 7(3):148–153. doi:10.1200/jop.2010.000106 21886493 10.1200/JOP.2010.000106 PMC3092652

[CR67] Tarone RE (1975) Tests for trend in life table analysis. Biometrika 62:679–682

[CR68] Tran B, Bedard PL (2011) Luminal-B breast cancer and novel therapeutic targets. Breast Cancer Res 13(6):221. doi:10.1186/bcr2904 22217398 10.1186/bcr2904 PMC3326541

[CR69] Untch M, Rezai M, Loibl S, Fasching PA, Huober J, Tesch H, Bauerfeind I, Hilfrich J, Eidtmann H, Gerber B, Hanusch C, Kuhn T, du Bois A, Blohmer JU, Thomssen C, Dan Costa S, Jackisch C, Kaufmann M, Mehta K, von Minckwitz G (2010) Neoadjuvant treatment with trastuzumab in HER2-positive breast cancer: results from the GeparQuattro study. J Clin Oncol 28(12):2024–2031. doi:10.1200/jco.2009.23.8451 20308670 10.1200/JCO.2009.23.8451

[CR70] Untch M, Fasching PA, Konecny GE, Hasmuller S, Lebeau A, Kreienberg R, Camara O, Muller V, du Bois A, Kuhn T, Stickeler E, Harbeck N, Hoss C, Kahlert S, Beck T, Fett W, Mehta KM, von Minckwitz G, Loibl S (2011) Pathologic complete response after neoadjuvant chemotherapy plus trastuzumab predicts favorable survival in human epidermal growth factor receptor 2-overexpressing breast cancer: results from the TECHNO trial of the AGO and GBG study groups. J Clin Oncol 29(25):3351–3357. doi:10.1200/jco.2010.31.4930 21788566 10.1200/JCO.2010.31.4930

[CR71] Untch M, Loibl S, Bischoff J, Eidtmann H, Kaufmann M, Blohmer JU, Hilfrich J, Strumberg D, Fasching PA, Kreienberg R, Tesch H, Hanusch C, Gerber B, Rezai M, Jackisch C, Huober J, Kuhn T, Nekljudova V, von Minckwitz G (2012) Lapatinib versus trastuzumab in combination with neoadjuvant anthracycline-taxane-based chemotherapy (GeparQuinto, GBG 44): a randomised phase 3 trial. Lancet Oncol 13(2):135–144. doi:10.1016/s1470-2045(11)70397-7 22257523 10.1016/S1470-2045(11)70397-7

[CR72] Valachis A, Mauri D, Polyzos NP, Chlouverakis G, Mavroudis D, Georgoulias V (2011) Trastuzumab combined to neoadjuvant chemotherapy in patients with HER2-positive breast cancer: a systematic review and meta-analysis. Breast 20(6):485–490. doi:10.1016/j.breast.2011.06.009 21784637 10.1016/j.breast.2011.06.009

[CR73] van der Hage JA, van de Velde CJ, Julien JP, Tubiana-Hulin M, Vandervelden C, Duchateau L (2001) Preoperative chemotherapy in primary operable breast cancer: results from the European Organization for Research and Treatment of Cancer trial 10902. J Clin Oncol 19(22):4224–4237 11709566 10.1200/JCO.2001.19.22.4224

[CR74] von Minckwitz G, Rezai M, Loibl S, Fasching PA, Huober J, Tesch H, Bauerfeind I, Hilfrich J, Eidtmann H, Gerber B, Hanusch C, Kuhn T, du Bois A, Blohmer JU, Thomssen C, Dan Costa S, Jackisch C, Kaufmann M, Mehta K, Untch M (2010) Capecitabine in addition to anthracycline- and taxane-based neoadjuvant treatment in patients with primary breast cancer: phase III GeparQuattro study. J Clin Oncol 28(12):2015–2023. doi:10.1200/jco.2009.23.8303 20308671 10.1200/JCO.2009.23.8303

[CR75] von Minckwitz G, Untch M, Nuesch E, Loibl S, Kaufmann M, Kummel S, Fasching PA, Eiermann W, Blohmer JU, Costa SD, Mehta K, Hilfrich J, Jackisch C, Gerber B, du Bois A, Huober J, Hanusch C, Konecny G, Fett W, Stickeler E, Harbeck N, Muller V, Juni P (2011) Impact of treatment characteristics on response of different breast cancer phenotypes: pooled analysis of the German neo-adjuvant chemotherapy trials. Breast Cancer Res Treat 125(1):145–156. doi:10.1007/s10549-010-1228-x 21042932 10.1007/s10549-010-1228-x

[CR76] von Minckwitz G, Untch M, Blohmer JU, Costa SD, Eidtmann H, Fasching PA, Gerber B, Eiermann W, Hilfrich J, Huober J, Jackisch C, Kaufmann M, Konecny GE, Denkert C, Nekljudova V, Mehta K, Loibl S (2012) Definition and impact of pathologic complete response on prognosis after neoadjuvant chemotherapy in various intrinsic breast cancer subtypes. J Clin Oncol 30(15):1796–1804. doi:10.1200/jco.2011.38.8595 22508812 10.1200/JCO.2011.38.8595

[CR77] Wang Y, Li JJ, Di GH, Lu JS, Wu J, Liu GY, Hu XC, Wang ZH, Yang WT, Shao ZM (2010) Retrospective analysis of trastuzumab treatment in 141 patients with Her-2 positive breast cancer. Zhonghua Zhong Liu Za Zhi 32(11):864–867 21223695

[CR78] Wildiers H, Neven P, Christiaens MR, Squifflet P, Amant F, Weltens C, Smeets A, van Limbergen E, Debrock G, Renard V, Van Eenoo L, Wynendaele W, Paridaens R (2011) Neoadjuvant capecitabine and docetaxel (plus trastuzumab): an effective non-anthracycline-based chemotherapy regimen for patients with locally advanced breast cancer. Ann Oncol 22(3):588–594. doi:10.1093/annonc/mdq406 20709813 10.1093/annonc/mdq406

